# Supplementary material for: Promoters adopt distinct dynamic manifestations depending on transcription factor context
Source: Mol Syst Biol. 2021 Feb 17;17(2):e9821. doi: 10.15252/msb.20209821 (PMC7888307; doi:10.15252/msb.20209821)
Supplement: Supplementary file 1 — Expanded View Figures PDF [file MSB-17-e9821-s001.pdf]

Expanded View Figures

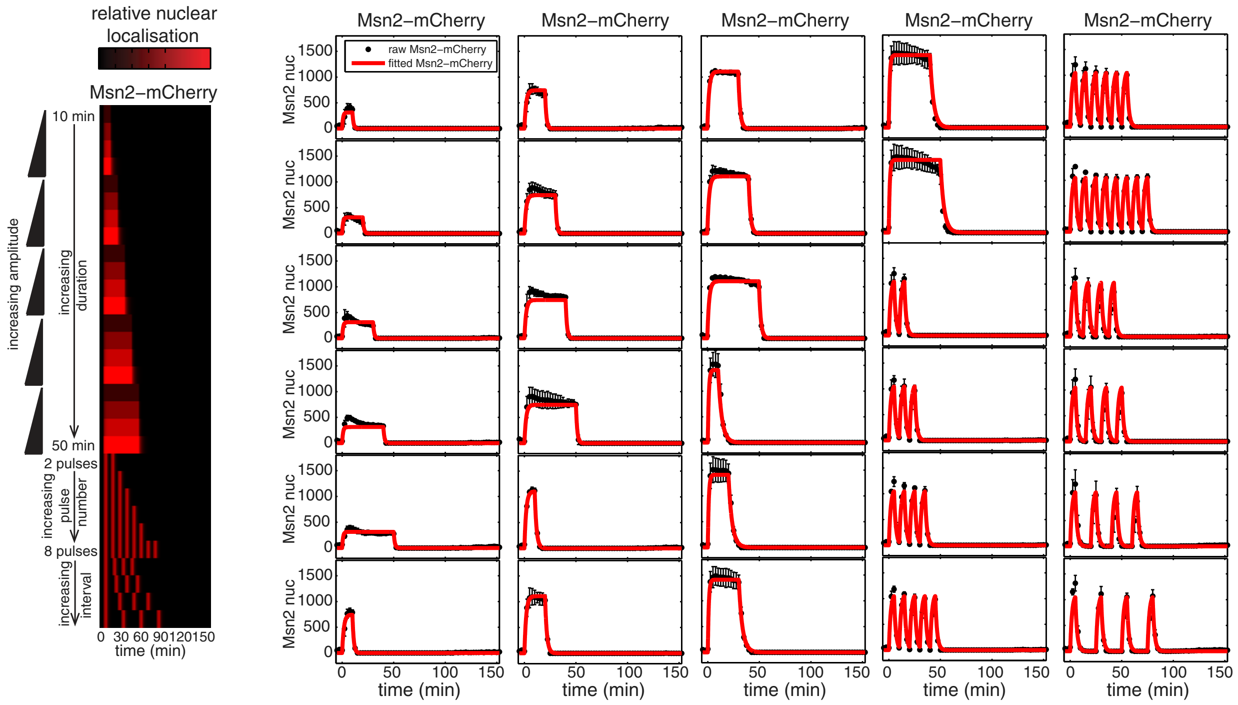

Figure EV1. Overview of Msn2 input experiments.
Left: heatmap overview of the 30 different Msn2-mCherry input. Right: Raw experimentally measured Msn2-mCherry input (black) and standard deviation (black error bars) for each of the 30 Msn2 inputs. The fitted Msn2-mCherry input is overlaid in red. This figure has been partially reproduced with permission from *Molecular Systems Biology* (Hansen & O'Shea, 2013).

Figure EV2. Evaluation of the inference method.

A, B Evaluation of the hybrid SMC method using synthetic data. Inference was performed using artificially generated time-course data as described in Methods and Protocols: Quantitative characterization of promoter dynamics. (A) Inference results for a promoter model with slow switching kinetics. (B) Inference results for a promoter model with fast-switching kinetics. Respective top panels show the results assuming perfect knowledge of the model parameters. Bottom panels show the corresponding results for random parameter mismatch, where each parameter was drawn from a log-normal distribution $LN(\log(b), 0.1^2)$ for each reconstruction, with b as the true value of the parameter. The R^2 and slope k between the true and predicted features were determined using linear regression (red dashed lines).

C, D Evaluation of the model calibration using moment-based inference. (C) Example fits are shown for some promoters for the 50 min pulse condition with 100% Msn2 induction level for one of the five independent inference runs. The models were calibrated using 50% of the trajectories and compared against means and variances calculated from the remaining pool of cells (i.e., those that were not used for fitting). Solid lines indicate averages and shaded areas mark one standard deviation above and below the average. (D) Quantitative analysis of the calibrated models across all conditions. To analyze the accuracy of the models, we calculated the absolute mismatch between the predicted and experimental means and variances at each time point. The resulting values were divided by the standard error of the experimental moments that we obtained using bootstrapping. Subsequently, we averaged these values across all time points and all five inference repeats. The resulting error statistic measures the accuracy relative to the uncertainty of the moment estimates obtained from data. A value close to one would indicate that the mismatch between the moments is comparable to the uncertainty of the experimental moments. The leftmost panel in (D) shows density histograms of the error statistic (err) for the mean and variance, respectively. The second and third panels show the correlation between the error statistic and the maximum of the average YFP level for each condition. The fourth and fifth panels show a cumulative error statistic for each promoter, calculated as the median across all conditions.

E, F Evaluation of the trajectory inference (C, D). To validate the reconstructed promoter switching and transcription dynamics, we calculated a maximum a posterior reconstruction of the time-varying transcription rate $Z(t)$ for all cells that were used for trajectory inference (i.e., 50% of the total pool of cells). These rates were then used to forward-simulate means and variances of YFP in combination with the calibrated parameters ω. The resulting means and variances were compared against the remaining pool of single-cell measurements, which were not used for trajectory inference as in (C, D). In general, the error statistics indicate a relatively good accuracy of the method. Larger errors were predominantly found for conditions with low YFP output (less than several hundred copies).

▶

© 2021 The Authors

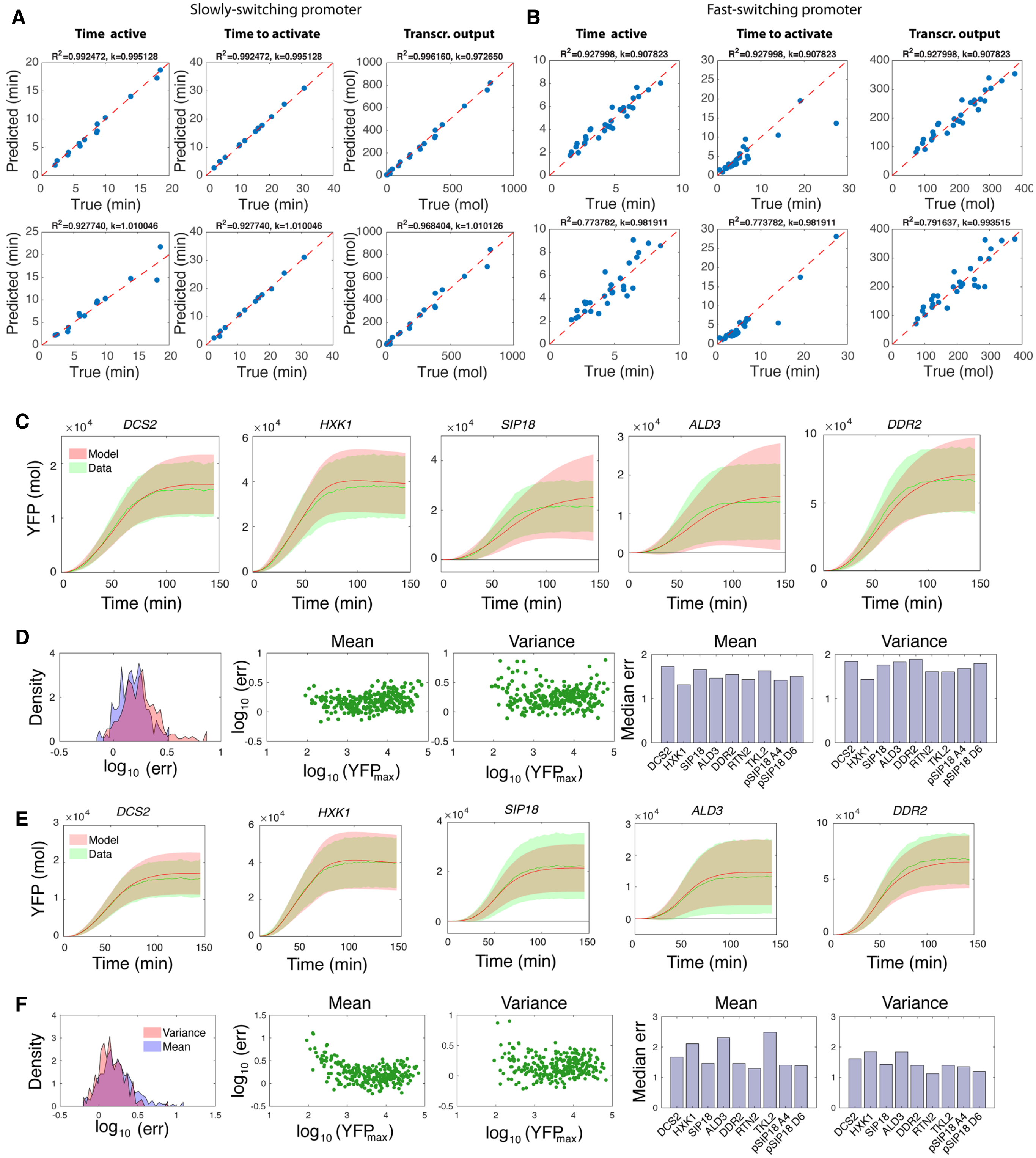

Figure EV2.

 © 2021 The Authors

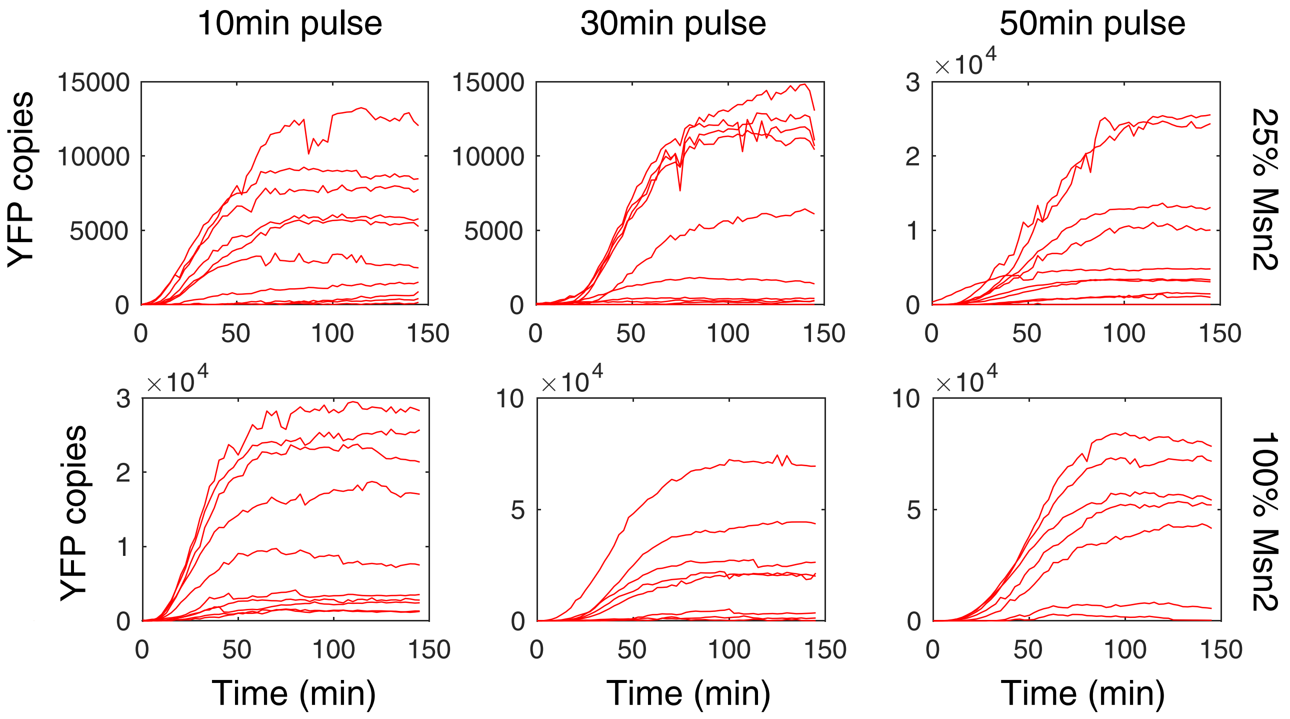

Figure EV3. Example single-cell trajectories for *DDR2* for the 10, 30, and 50 min pulse conditions with 25 and 100% Msn2 induction level, respectively.

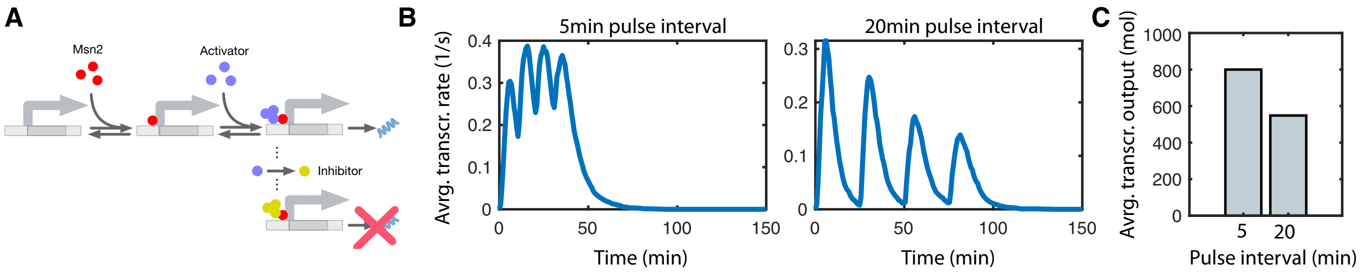

Figure EV4. Toy model with interval-dependent promoter memory.

A Model scheme. Once Msn2 binds to the promoter, activator molecules can be recruited, which causes the promoter to switch into a transcriptionally active state with a rate proportional to the number of activators present. Once the promoter switches back into the Msn2-unbound state, the activator can be converted into an inhibitor, which causes the promoter to switch into a transcriptionally inactive state with a rate proportional to the number of inhibitors present.

B Average transcription rate for 5 and 20 min pulse intervals as a function of time obtained by forward simulation of the model. Blue lines indicate averages computed from stochastic simulations (n = 2,000).

C Corresponding average transcriptional output for 5 and 20 min pulse intervals. A detailed reaction scheme and parameters used for simulation can be found in Methods and Protocols: Toy model of interval-dependent promoter memory. We emphasize that this toy model only serves to illustrate one possible scenario, which could result in a pulse interval-dependent switch from positive to negative memory, as observed in Fig 4A. We do not currently understand the mechanism underlying the observation in Fig 4A.

© 2021 The Authors

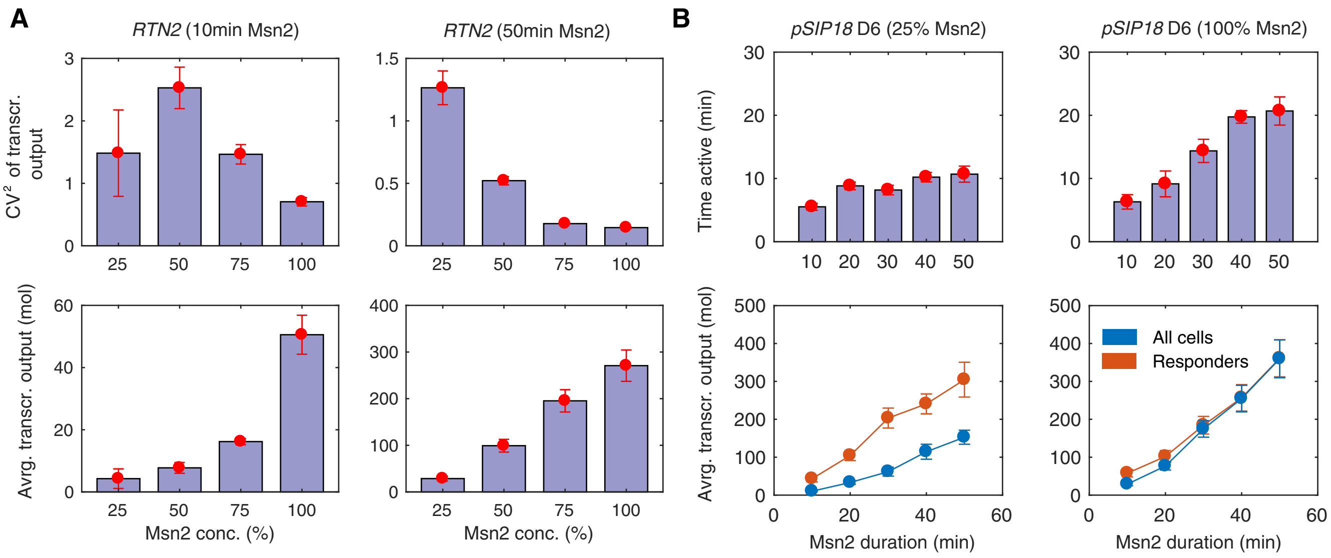

Figure EV5. Additional examples of curious behaviors and potential manifestations.

A For *RTN2*, the noise of transcriptional output shows a non-monotonic scaling with Msn2 amplitude for 10 min Msn2 single-pulse duration, but a monotonically decreasing relationship for 50 min Msn2 duration. The average transcriptional output increases monotonically with Msn2 amplitude for both durations.

B For *SIP18* mutant D6, time active is largely independent of pulse length for 25% Msn2 amplitude but increases with pulse length for higher Msn2 amplitudes. The transcriptional output averaged over all cells (bottom panel, blue) increases with pulse length and Msn2 amplitude. However, the average transcriptional output of only responding cells is very similar for both Msn2 amplitudes. The examples shown in (A, B) illustrate further interesting behaviors and potential manifestations, but we emphasize that more analysis will be required to validate the robustness of these results.

© 2021 The Authors
